# Crystal structure of di­ethyl­ammonium aniline-4-sulfonate anilinium-4-sulfonate

**DOI:** 10.1107/S2056989016018041

**Published:** 2016-11-18

**Authors:** Assane Toure, Libasse Diop, Cheikh Abdoul Khadir Diop, Allen G. Oliver

**Affiliations:** a Laboratoire de Chimie Minérale et Analytique, Département de Chimie, Faculté des Sciences et Techniques, Université Cheikh Anta Diop, Dakar, Senegal; b Department of Chemistry and Biochemistry, University of Notre Dame, 246 Nieuwland, Science Hall, Notre Dame, IN 46557-5670, USA

**Keywords:** crystal structure, di­ethyl­ammonium cation, aniline­sulfonic zwitterion, aniline­sulfonate, hydrogen bonds, three-dimensional structure

## Abstract

The title compound, C_4_H_12_N^+^·C_6_H_6_NO_3_S^−^·C_6_H_7_NO_3_S, consists of an ion pair and a zwitterionic neutral mol­ecule. The cation adopts an extended conformation [C—C—N—C torsion angles = 177.1 (3) and −178.4 (3)°]. In the crystal, the components are linked by N—H⋯O and N—H⋯N hydrogen bonds, generating a three-dimensional network, which is consolidated by weak C—H⋯O inter­actions.

## Abstract 2

The title compound consists of an di­ethyl­ammonium–aniline­sulfonate ion pair and a zwitterionic aniliniumsulfonate mol­ecule.

## Chemical context   

Acids such as sulfuric, nitric, oxalic, phospho­ric, substituted sulfonic, etc. when mixed in water with amines give acidic or neutral salts that may be soluble in organic solvents: this solubility allows for the study of their inter­actions with metal halides, acetates, nitrates, perchlorates, *etc*, which yield new adducts and complexes in which the conjugate anion of the acid behaves as a ligand, usually coordinating the metal ion (Najafi *et al.*, 2011*a* ▸,*b* ▸; Ittyachan *et al.*, 2016 ▸; Majeed & Wendt, 2016 ▸).
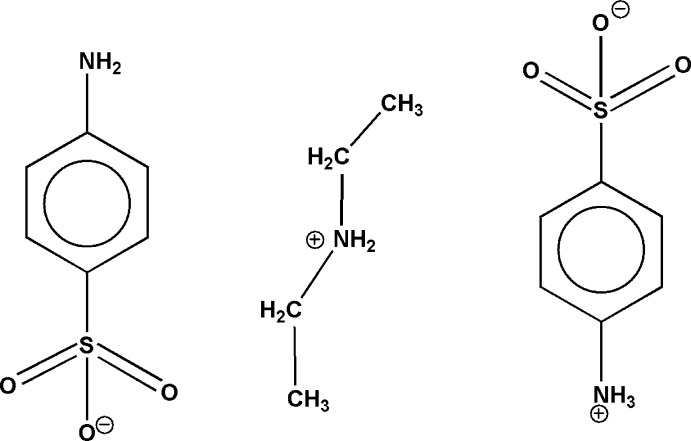


We report here the synthesis and structure of the product arising from the mixing of di­ethyl­amine and aniline­sulfonic acid solutions, which contains a combination of ions and a zwitterion. In terms of other compounds containing both the aniline­sulfonate anion and its zwitterionic form, anilinium­sulfonate, to date only the 4-amino­pyridinium salt has been reported (Fun *et al.*, 2008 ▸).

## Structural commentary   

**Figure 1:**
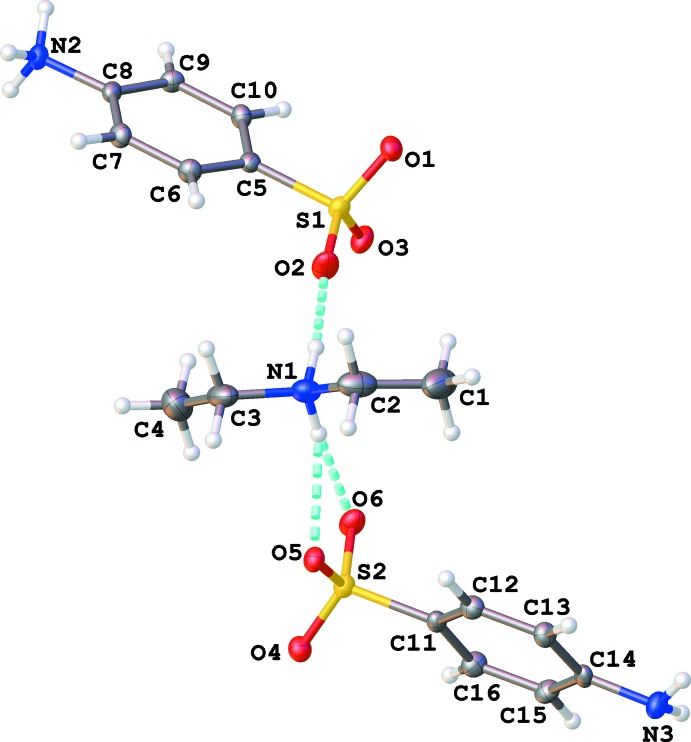
The mol­ecular structure of the title compound. Displacement ellipsoids depicted at the 50% probability level and H atoms as spheres of an arbitrary radius. Hydrogen bonds are represented by light-blue dashed lines.

There is one di­ethyl­ammonium cation, one aniline­sulfonate anion and one zwitterionic aniliniumsulfonate mol­ecule in the asymmetric unit (Fig. 1 ▸). The individual mol­ecules are unremarkable with bond distances and angles typical of their type. The cation adopts an extended conformation [C1—C2—N1—C3 and C2—N1—C3—C4 torsion angles = 177.1 (3) and −178.4 (3)°, respectively].

## Supra­molecular features   

**Figure 2:**
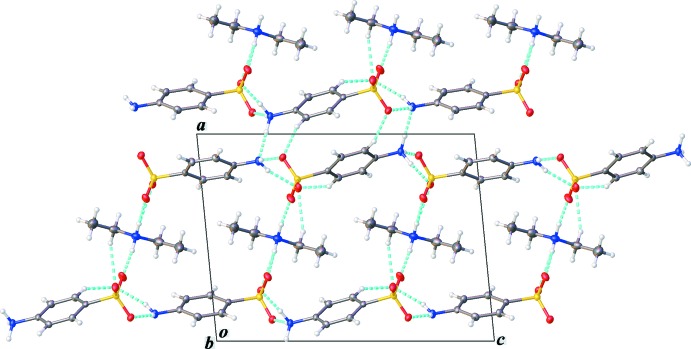
Packing diagram, viewed along the *b* axis. Hydrogen bonds are represented by light-blue dashed lines.

The zwitterionic aniliniumsulfonate and the aniline­sulfonate anion are connected through N2—H2*NA*⋯O4^i^, N2—H2*NB*⋯O5^ii^, N2—H2*NC*⋯N3^iii^, N3—H3*NA*⋯O1^iv^ and N3—H3*NB*⋯O3^v^ hydrogen bonds (Table 1 ▸) giving sheet-like bi-layers that lie parallel to the *bc* plane [symmetry codes: (i) −*x* + 1, *y* − 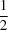, −*z* + 1; (ii) −*x* + 1, *y* + 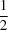, −*z* + 1; (iii) *x* − 1, *y*, *z* − 1; (iv) −*x* + 1, *y* + 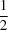, −*z* + 2; (v) −*x* + 1, *y* − 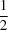, −*z* + 2]. The bi-layers are then linked through N1—H1*NA*⋯O2, N1—H1*NB*⋯O5 and N1—H1*NB*⋯O6 hydrogen bonds, yielding a three-dimensional network (Fig. 2 ▸). Some weak C—H⋯O (C3—H3*A*⋯O3^vi^, C6—H6⋯O3^vi^ and C9—H9⋯O1^vii^) inter­actions consolidate the packing in the crystal [symmetry codes: (vi) *x*, *y* − 1, *z*; (vii) −*x*, *y* + 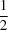, −*z* + 1]. Examination of the packing reveals layers of diethyl ammonium cation sandwiched between bi-layers of aniline sulfate moieties. The key hydrogen bonds establishing the three-dimensional array are the contacts to sulfonate oxygen atoms and the N2⋯N3 aniline inter­actions. All amine hydrogen atoms form good hydrogen-bond contacts to neighboring hydrogen-bond acceptor atoms.

## Database survey   

A search of the Cambridge Structural Database (Version 5.37 + one update; Groom *et al.*, 2016 ▸) shows 46 hits concerning the aniline­sulfonate anion, three containing aniliniumsulfonate and one hit with both (Fun *et al.*, 2008 ▸), while 303 hits concern the di­ethyl­ammonium ion.

## Synthesis and crystallization   

Dimethyl amine was mixed in water with aniline sulfonic acid in a 1:1 ratio. Colorless block-like crystals were obtained on allowing the water to evaporate at 333 K.

## Refinement   

Crystal data, data collection and structure refinement details are summarized in Table 2 ▸. Hydrogen atoms bonded to carbon were included in geometrically calculated positions and allowed to ride on the parent atom. All amine hydrogen atoms were located in a difference Fourier map and refined freely.

As the mol­ecules are achiral, only the correct enanti­omorph of the space group was determined: this was determined by comparison of intensities of Friedel pairs of reflections yielding a Flack *x* parameter of 0.03 (6) (Parsons *et al.*, 2013 ▸) and a Hooft *y* parameter of 0.04 (6) (Hooft *et al.*, 2008 ▸).

## Supplementary Material

Crystal structure: contains datablock(s) I. DOI: 10.1107/S2056989016018041/hb7622sup1.cif

Structure factors: contains datablock(s) I. DOI: 10.1107/S2056989016018041/hb7622Isup2.hkl

Supporting information file. DOI: 10.1107/S2056989016018041/hb7622Isup3.cml

CCDC reference: 1515845

Additional supporting information: crystallographic information; 3D view; checkCIF report

## Figures and Tables

**Table 1:** Hydrogen-bond geometry (Å, °)

*D*—H⋯*A*	*D*—H	H⋯*A*	*D*⋯*A*	*D*—H⋯*A*
N1—H1*NA*⋯O2	0.96 (3)	1.79 (4)	2.748 (4)	175 (3)
N1—H1*NB*⋯O5	0.90 (3)	2.45 (3)	3.019 (4)	122 (3)
N1—H1*NB*⋯O6	0.90 (3)	2.03 (4)	2.920 (4)	173 (3)
N2—H2*NA*⋯O4^i^	0.94 (2)	1.88 (2)	2.794 (4)	165 (3)
N2—H2*NB*⋯O5^ii^	0.94 (2)	1.85 (2)	2.778 (3)	171 (3)
N2—H2*NC*⋯N3^iii^	0.97 (2)	1.85 (2)	2.812 (4)	178 (4)
N3—H3*NA*⋯O1^iv^	0.83 (4)	2.21 (4)	2.998 (4)	157 (3)
N3—H3*NB*⋯O3^v^	0.79 (3)	2.23 (3)	2.997 (4)	164 (3)
C3—H3*A*⋯O3^vi^	0.99	2.48	3.338 (4)	145
C6—H6⋯O3^vi^	0.95	2.65	3.507 (4)	151
C9—H9⋯O1^vii^	0.95	2.59	3.517 (4)	165

Symmetry codes: (i) 
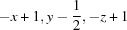
; (ii) 
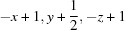
; (iii) 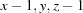; (iv) 
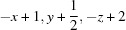
; (v) 
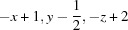
; (vi) 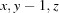; (vii) 
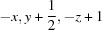
.

**Table 2:** Experimental details

Crystal data	
Chemical formula	C_4_H_12_N^+^·C_6_H_6_NO_3_S^−^·C_6_H_7_NO_3_S
*M*_r_	419.51
Crystal system, space group	Monoclinic, *P*2_1_
Temperature (K)	120
*a*, *b*, *c* (Å)	11.419 (3), 5.6731 (16), 15.226 (4)
β (°)	95.530 (4)
*V* (Å^3^)	981.8 (5)
*Z*	2
Radiation type	Mo *K*α
μ (mm^−1^)	0.31
Crystal size (mm)	0.22 × 0.19 × 0.05

Data collection	
Diffractometer	Bruker APEXII CCD
Absorption correction	Multi-scan (*SADABS*; Krause *et al.*, 2015 ▸)
*T*_min_, *T*_max_	0.781, 0.931
No. of measured, independent and observed [*I* > 2σ(*I*)] reflections	18906, 4911, 4228
*R*_int_	0.056
(sin θ/λ)_max_ (Å^−1^)	0.668

Refinement	
*R*[*F* ^2^ > 2σ(*F* ^2^)], *wR*(*F* ^2^), *S*	0.039, 0.077, 0.98
No. of reflections	4911
No. of parameters	274
No. of restraints	4
H-atom treatment	H atoms treated by a mixture of independent and constrained refinement
Δρ_max_, Δρ_min_ (e Å^−3^)	0.29, −0.38
Absolute structure	Flack *x* determined using 1632 quotients [(*I* ^+^)−(*I* ^−^)]/[(*I* ^+^)+(*I* ^−^)] (Parsons *et al.*, 2013 ▸)
Absolute structure parameter	0.03 (6)

Computer programs: *APEX3* and *SAINT* (Bruker, 2015 ▸), *SHELXT2014* (Sheldrick, 2015*a* ▸), *SHELXL2014* (Sheldrick, 2015*b* ▸), *OLEX2* (Dolomanov *et al.*, 2009 ▸) and *publCIF* (Westrip, 2010 ▸).

## supplementary crystallographic information

### Crystal data

**Table:**

C_4_H_12_N^+^·C_6_H_6_NO_3_S^−^·C_6_H_7_NO_3_S	*F*(000) = 444
*M**_r_* = 419.51	*D*_x_ = 1.419 Mg m^−^^3^
Monoclinic, *P*2_1_	Mo *K*α radiation, λ = 0.71073 Å
*a* = 11.419 (3) Å	Cell parameters from 4341 reflections
*b* = 5.6731 (16) Å	θ = 2.7–24.5°
*c* = 15.226 (4) Å	µ = 0.31 mm^−^^1^
β = 95.530 (4)°	*T* = 120 K
*V* = 981.8 (5) Å^3^	Plate, colorless
*Z* = 2	0.22 × 0.19 × 0.05 mm

### Data collection

**Table:**

Bruker APEXII CCD diffractometer	4911 independent reflections
Radiation source: fine-focus sealed tube	4228 reflections with *I* > 2σ(*I*)
Graphite monochromator	*R*_int_ = 0.056
Detector resolution: 8.33 pixels mm^-1^	θ_max_ = 28.4°, θ_min_ = 1.3°
combination of ω and φ–scans	*h* = −15→15
Absorption correction: multi-scan (SADABS; Krause *et al.*, 2015)	*k* = −7→7
*T*_min_ = 0.781, *T*_max_ = 0.931	*l* = −20→20
18906 measured reflections	

### Refinement

**Table:**

Refinement on *F*^2^	Secondary atom site location: difference Fourier map
Least-squares matrix: full	Hydrogen site location: mixed
*R*[*F*^2^ > 2σ(*F*^2^)] = 0.039	H atoms treated by a mixture of independent and constrained refinement
*wR*(*F*^2^) = 0.077	*w* = 1/[σ^2^(*F*_o_^2^) + (0.0303*P*)^2^] where *P* = (*F*_o_^2^ + 2*F*_c_^2^)/3
*S* = 0.98	(Δ/σ)_max_ = 0.001
4911 reflections	Δρ_max_ = 0.29 e Å^−^^3^
274 parameters	Δρ_min_ = −0.38 e Å^−^^3^
4 restraints	Absolute structure: Flack *x* determined using 1632 quotients [(*I*^+^)-(*I*^-^)]/[(*I*^+^)+(*I*^-^)] (Parsons *et al.*, 2013)
Primary atom site location: real-space vector search	Absolute structure parameter: 0.03 (6)

### Special details

**Table:**

Geometry. All esds (except the esd in the dihedral angle between two l.s. planes) are estimated using the full covariance matrix. The cell esds are taken into account individually in the estimation of esds in distances, angles and torsion angles; correlations between esds in cell parameters are only used when they are defined by crystal symmetry. An approximate (isotropic) treatment of cell esds is used for estimating esds involving l.s. planes.

### Fractional atomic coordinates and isotropic or equivalent isotropic displacement parameters (Å^2^)

**Table:**

	*x*	*y*	*z*	*U*_iso_*/*U*_eq_	
N1	0.5053 (3)	0.0636 (5)	0.74539 (18)	0.0247 (7)	
H1NA	0.437 (3)	0.156 (6)	0.727 (2)	0.033 (10)*	
H1NB	0.566 (3)	0.160 (6)	0.761 (2)	0.031 (11)*	
C1	0.4529 (3)	0.0950 (8)	0.8976 (2)	0.0461 (11)	
H1A	0.4323	0.0039	0.9486	0.069*	
H1B	0.3869	0.1973	0.8767	0.069*	
H1C	0.5224	0.1918	0.9148	0.069*	
C2	0.4791 (3)	−0.0698 (7)	0.8253 (2)	0.0365 (10)	
H2A	0.5474	−0.1696	0.8458	0.044*	
H2B	0.4106	−0.1743	0.8104	0.044*	
C3	0.5386 (3)	−0.0861 (7)	0.6708 (2)	0.0336 (9)	
H3A	0.4730	−0.1938	0.6512	0.040*	
H3B	0.6083	−0.1829	0.6907	0.040*	
C4	0.5661 (3)	0.0694 (8)	0.5950 (2)	0.0459 (11)	
H4A	0.5848	−0.0291	0.5453	0.069*	
H4B	0.6337	0.1699	0.6138	0.069*	
H4C	0.4977	0.1683	0.5767	0.069*	
S1	0.21877 (6)	0.48653 (13)	0.65278 (5)	0.01702 (18)	
O1	0.11341 (17)	0.4576 (4)	0.69828 (12)	0.0192 (5)	
O2	0.3108 (2)	0.3205 (4)	0.68312 (15)	0.0281 (6)	
O3	0.25973 (19)	0.7296 (4)	0.65346 (13)	0.0221 (5)	
N2	0.0854 (2)	0.2713 (5)	0.27136 (16)	0.0166 (6)	
H2NA	0.088 (3)	0.109 (4)	0.2598 (18)	0.017 (8)*	
H2NB	0.144 (2)	0.338 (6)	0.239 (2)	0.027 (10)*	
H2NC	0.010 (2)	0.341 (7)	0.252 (2)	0.051 (12)*	
C5	0.1782 (3)	0.4162 (6)	0.54039 (19)	0.0152 (7)	
C6	0.2099 (3)	0.2003 (5)	0.50528 (19)	0.0180 (7)	
H6	0.2527	0.0874	0.5416	0.022*	
C7	0.1781 (3)	0.1530 (6)	0.41663 (19)	0.0181 (7)	
H7	0.1995	0.0075	0.3917	0.022*	
C8	0.1153 (3)	0.3183 (5)	0.36490 (19)	0.0149 (7)	
C9	0.0805 (3)	0.5306 (5)	0.39992 (19)	0.0162 (6)	
H9	0.0359	0.6415	0.3639	0.019*	
C10	0.1122 (3)	0.5774 (5)	0.48805 (19)	0.0161 (7)	
H10	0.0886	0.7213	0.5130	0.019*	
S2	0.78723 (7)	0.26125 (13)	0.83062 (5)	0.01669 (18)	
O4	0.89636 (19)	0.3121 (4)	0.79229 (13)	0.0194 (5)	
O5	0.75682 (17)	0.0109 (4)	0.82423 (12)	0.0173 (5)	
O6	0.68960 (19)	0.4094 (4)	0.79409 (14)	0.0231 (5)	
N3	0.8696 (3)	0.4851 (6)	1.21318 (17)	0.0194 (6)	
H3NA	0.874 (3)	0.630 (7)	1.223 (2)	0.028 (11)*	
H3NB	0.824 (3)	0.430 (6)	1.244 (2)	0.020 (10)*	
C11	0.8122 (3)	0.3252 (5)	0.94423 (19)	0.0146 (7)	
C12	0.7796 (3)	0.1654 (5)	1.00663 (19)	0.0181 (7)	
H12	0.7445	0.0197	0.9881	0.022*	
C13	0.7982 (3)	0.2179 (5)	1.09560 (18)	0.0178 (7)	
H13	0.7767	0.1071	1.1380	0.021*	
C14	0.8485 (3)	0.4330 (5)	1.12326 (19)	0.0159 (7)	
C15	0.8832 (3)	0.5891 (5)	1.06041 (19)	0.0190 (7)	
H15	0.9198	0.7337	1.0787	0.023*	
C16	0.8650 (3)	0.5360 (5)	0.9717 (2)	0.0201 (7)	
H16	0.8889	0.6444	0.9293	0.024*	

### Atomic displacement parameters (Å^2^)

**Table:**

	*U*^11^	*U*^22^	*U*^33^	*U*^12^	*U*^13^	*U*^23^
N1	0.0186 (16)	0.0267 (17)	0.0282 (16)	0.0017 (13)	−0.0008 (13)	0.0020 (13)
C1	0.033 (2)	0.077 (3)	0.029 (2)	−0.013 (2)	0.0030 (18)	0.004 (2)
C2	0.0242 (19)	0.043 (3)	0.041 (2)	−0.0057 (18)	−0.0012 (17)	0.0136 (19)
C3	0.0192 (19)	0.039 (2)	0.042 (2)	0.0001 (16)	−0.0021 (16)	−0.0131 (18)
C4	0.034 (2)	0.068 (3)	0.036 (2)	0.005 (2)	0.0077 (19)	−0.006 (2)
S1	0.0176 (4)	0.0183 (4)	0.0148 (4)	0.0014 (3)	−0.0006 (3)	−0.0003 (3)
O1	0.0206 (12)	0.0203 (13)	0.0174 (11)	−0.0024 (10)	0.0057 (9)	−0.0005 (10)
O2	0.0288 (14)	0.0326 (15)	0.0214 (12)	0.0145 (11)	−0.0059 (10)	−0.0002 (10)
O3	0.0275 (13)	0.0213 (13)	0.0176 (11)	−0.0072 (11)	0.0022 (9)	−0.0037 (10)
N2	0.0222 (15)	0.0139 (14)	0.0139 (13)	−0.0004 (13)	0.0023 (11)	−0.0005 (12)
C5	0.0141 (16)	0.0172 (17)	0.0147 (15)	−0.0027 (13)	0.0027 (12)	0.0000 (12)
C6	0.0194 (17)	0.0157 (16)	0.0186 (15)	0.0026 (13)	0.0008 (13)	0.0012 (12)
C7	0.0203 (17)	0.0145 (15)	0.0198 (16)	0.0004 (13)	0.0030 (14)	−0.0026 (13)
C8	0.0153 (16)	0.0178 (17)	0.0120 (15)	−0.0033 (12)	0.0029 (12)	−0.0001 (12)
C9	0.0169 (15)	0.0143 (16)	0.0172 (15)	0.0007 (12)	0.0000 (12)	0.0037 (12)
C10	0.0195 (17)	0.0131 (15)	0.0162 (15)	0.0003 (12)	0.0037 (13)	−0.0008 (12)
S2	0.0195 (4)	0.0154 (4)	0.0149 (4)	0.0007 (3)	0.0008 (3)	−0.0002 (3)
O4	0.0218 (12)	0.0188 (13)	0.0182 (11)	−0.0011 (10)	0.0047 (9)	0.0008 (9)
O5	0.0218 (11)	0.0144 (11)	0.0161 (10)	−0.0019 (10)	0.0027 (9)	−0.0019 (9)
O6	0.0261 (13)	0.0218 (13)	0.0201 (12)	0.0053 (10)	−0.0037 (10)	−0.0001 (9)
N3	0.0260 (16)	0.0171 (15)	0.0152 (13)	−0.0031 (14)	0.0023 (12)	−0.0007 (13)
C11	0.0155 (16)	0.0155 (17)	0.0127 (15)	0.0033 (12)	0.0011 (12)	0.0009 (12)
C12	0.0189 (17)	0.0142 (15)	0.0213 (16)	−0.0004 (13)	0.0020 (13)	−0.0005 (12)
C13	0.0215 (17)	0.0162 (16)	0.0162 (15)	−0.0003 (13)	0.0037 (13)	0.0025 (12)
C14	0.0170 (16)	0.0167 (17)	0.0141 (15)	0.0053 (13)	0.0022 (12)	−0.0019 (12)
C15	0.0206 (17)	0.0144 (16)	0.0214 (16)	−0.0019 (13)	−0.0009 (14)	−0.0016 (13)
C16	0.0228 (17)	0.0183 (18)	0.0191 (16)	−0.0019 (13)	0.0019 (13)	0.0034 (13)

### Geometric parameters (Å, º)

**Table:**

N1—C2	1.488 (4)	C6—C7	1.390 (4)
N1—C3	1.497 (4)	C6—H6	0.9500
N1—H1NA	0.96 (3)	C7—C8	1.380 (4)
N1—H1NB	0.90 (3)	C7—H7	0.9500
C1—C2	1.496 (5)	C8—C9	1.390 (4)
C1—H1A	0.9800	C9—C10	1.381 (4)
C1—H1B	0.9800	C9—H9	0.9500
C1—H1C	0.9800	C10—H10	0.9500
C2—H2A	0.9900	S2—O4	1.455 (2)
C2—H2B	0.9900	S2—O6	1.462 (2)
C3—C4	1.510 (5)	S2—O5	1.463 (2)
C3—H3A	0.9900	S2—C11	1.763 (3)
C3—H3B	0.9900	N3—C14	1.399 (4)
C4—H4A	0.9800	N3—H3NA	0.83 (4)
C4—H4B	0.9800	N3—H3NB	0.79 (3)
C4—H4C	0.9800	C11—C16	1.386 (4)
S1—O2	1.453 (2)	C11—C12	1.389 (4)
S1—O1	1.454 (2)	C12—C13	1.383 (4)
S1—O3	1.456 (2)	C12—H12	0.9500
S1—C5	1.775 (3)	C13—C14	1.397 (4)
N2—C8	1.457 (4)	C13—H13	0.9500
N2—H2NA	0.94 (2)	C14—C15	1.389 (4)
N2—H2NB	0.94 (2)	C15—C16	1.380 (4)
N2—H2NC	0.97 (2)	C15—H15	0.9500
C5—C10	1.387 (4)	C16—H16	0.9500
C5—C6	1.398 (4)		
			
C2—N1—C3	114.7 (3)	C6—C5—S1	120.8 (2)
C2—N1—H1NA	107 (2)	C7—C6—C5	119.2 (3)
C3—N1—H1NA	110 (2)	C7—C6—H6	120.4
C2—N1—H1NB	108 (2)	C5—C6—H6	120.4
C3—N1—H1NB	107 (2)	C8—C7—C6	119.7 (3)
H1NA—N1—H1NB	109 (3)	C8—C7—H7	120.1
C2—C1—H1A	109.5	C6—C7—H7	120.1
C2—C1—H1B	109.5	C7—C8—C9	121.4 (3)
H1A—C1—H1B	109.5	C7—C8—N2	119.6 (3)
C2—C1—H1C	109.5	C9—C8—N2	119.0 (3)
H1A—C1—H1C	109.5	C10—C9—C8	118.8 (3)
H1B—C1—H1C	109.5	C10—C9—H9	120.6
N1—C2—C1	110.7 (3)	C8—C9—H9	120.6
N1—C2—H2A	109.5	C9—C10—C5	120.5 (3)
C1—C2—H2A	109.5	C9—C10—H10	119.7
N1—C2—H2B	109.5	C5—C10—H10	119.7
C1—C2—H2B	109.5	O4—S2—O6	112.61 (13)
H2A—C2—H2B	108.1	O4—S2—O5	111.89 (13)
N1—C3—C4	109.6 (3)	O6—S2—O5	111.41 (13)
N1—C3—H3A	109.7	O4—S2—C11	106.81 (14)
C4—C3—H3A	109.7	O6—S2—C11	107.45 (14)
N1—C3—H3B	109.7	O5—S2—C11	106.25 (14)
C4—C3—H3B	109.7	C14—N3—H3NA	112 (2)
H3A—C3—H3B	108.2	C14—N3—H3NB	115 (2)
C3—C4—H4A	109.5	H3NA—N3—H3NB	109 (3)
C3—C4—H4B	109.5	C16—C11—C12	119.6 (3)
H4A—C4—H4B	109.5	C16—C11—S2	119.9 (2)
C3—C4—H4C	109.5	C12—C11—S2	120.5 (2)
H4A—C4—H4C	109.5	C13—C12—C11	120.2 (3)
H4B—C4—H4C	109.5	C13—C12—H12	119.9
O2—S1—O1	112.42 (14)	C11—C12—H12	119.9
O2—S1—O3	112.94 (15)	C12—C13—C14	120.2 (3)
O1—S1—O3	112.55 (13)	C12—C13—H13	119.9
O2—S1—C5	105.94 (14)	C14—C13—H13	119.9
O1—S1—C5	106.42 (13)	C15—C14—C13	119.1 (3)
O3—S1—C5	105.88 (14)	C15—C14—N3	120.4 (3)
C8—N2—H2NA	110.7 (18)	C13—C14—N3	120.5 (3)
C8—N2—H2NB	109 (2)	C16—C15—C14	120.6 (3)
H2NA—N2—H2NB	105 (3)	C16—C15—H15	119.7
C8—N2—H2NC	110 (2)	C14—C15—H15	119.7
H2NA—N2—H2NC	113 (3)	C15—C16—C11	120.2 (3)
H2NB—N2—H2NC	109 (3)	C15—C16—H16	119.9
C10—C5—C6	120.3 (3)	C11—C16—H16	119.9
C10—C5—S1	118.9 (2)		
			
C3—N1—C2—C1	177.1 (3)	S1—C5—C10—C9	178.9 (2)
C2—N1—C3—C4	−178.4 (3)	O4—S2—C11—C16	47.7 (3)
O2—S1—C5—C10	−165.1 (2)	O6—S2—C11—C16	−73.4 (3)
O1—S1—C5—C10	75.1 (3)	O5—S2—C11—C16	167.2 (2)
O3—S1—C5—C10	−44.9 (3)	O4—S2—C11—C12	−132.1 (3)
O2—S1—C5—C6	16.1 (3)	O6—S2—C11—C12	106.9 (3)
O1—S1—C5—C6	−103.7 (3)	O5—S2—C11—C12	−12.5 (3)
O3—S1—C5—C6	136.3 (2)	C16—C11—C12—C13	1.0 (4)
C10—C5—C6—C7	2.2 (4)	S2—C11—C12—C13	−179.3 (2)
S1—C5—C6—C7	−179.0 (2)	C11—C12—C13—C14	0.8 (4)
C5—C6—C7—C8	−0.4 (4)	C12—C13—C14—C15	−2.3 (4)
C6—C7—C8—C9	−1.4 (5)	C12—C13—C14—N3	−178.8 (3)
C6—C7—C8—N2	178.0 (3)	C13—C14—C15—C16	2.0 (4)
C7—C8—C9—C10	1.4 (4)	N3—C14—C15—C16	178.5 (3)
N2—C8—C9—C10	−178.0 (3)	C14—C15—C16—C11	−0.2 (5)
C8—C9—C10—C5	0.5 (4)	C12—C11—C16—C15	−1.3 (4)
C6—C5—C10—C9	−2.3 (4)	S2—C11—C16—C15	179.0 (2)

### Hydrogen-bond geometry (Å, º)

**Table:**

*D*—H···*A*	*D*—H	H···*A*	*D*···*A*	*D*—H···*A*
N1—H1*NA*···O2	0.96 (3)	1.79 (4)	2.748 (4)	175 (3)
N1—H1*NB*···O5	0.90 (3)	2.45 (3)	3.019 (4)	122 (3)
N1—H1*NB*···O6	0.90 (3)	2.03 (4)	2.920 (4)	173 (3)
N2—H2*NA*···O4^i^	0.94 (2)	1.88 (2)	2.794 (4)	165 (3)
N2—H2*NB*···O5^ii^	0.94 (2)	1.85 (2)	2.778 (3)	171 (3)
N2—H2*NC*···N3^iii^	0.97 (2)	1.85 (2)	2.812 (4)	178 (4)
N3—H3*NA*···O1^iv^	0.83 (4)	2.21 (4)	2.998 (4)	157 (3)
N3—H3*NB*···O3^v^	0.79 (3)	2.23 (3)	2.997 (4)	164 (3)
C3—H3*A*···O3^vi^	0.99	2.48	3.338 (4)	145
C6—H6···O3^vi^	0.95	2.65	3.507 (4)	151
C9—H9···O1^vii^	0.95	2.59	3.517 (4)	165

Symmetry codes: (i) −*x*+1, *y*−1/2, −*z*+1; (ii) −*x*+1, *y*+1/2, −*z*+1; (iii) *x*−1, *y*, *z*−1; (iv) −*x*+1, *y*+1/2, −*z*+2; (v) −*x*+1, *y*−1/2, −*z*+2; (vi) *x*, *y*−1, *z*; (vii) −*x*, *y*+1/2, −*z*+1.
